# Serum N-Acetylneuraminic Acid Is Associated with Atrial Fibrillation and Left Atrial Enlargement

**DOI:** 10.1155/2020/1358098

**Published:** 2020-04-13

**Authors:** Wei Hu, Jing Xie, Tongjian Zhu, Guannan Meng, Meng Wang, Zhen Zhou, Fuding Guo, Hui Chen, Zhuo Wang, Songyun Wang, Huafen Liu, Hong Jiang

**Affiliations:** Department of Cardiology, Renmin Hospital of Wuhan University, Cardiovascular Research Institute, Wuhan University, Hubei Key Laboratory of Cardiology, Wuhan, Hubei 430060, China

## Abstract

**Purpose:**

Recent studies have indicated that N-acetylneuraminic acid (Neu5Ac) plays a key role in severe coronary artery diseases, involving RhoA signaling pathway activation, which is critically involved in cardiac fibrosis. There is convincing evidence from many studies that left atrium fibrosis is involved in the pathophysiology of AF. Therefore, we speculated that Neu5Ac may be associated with atrial fibrillation (AF) and involved in the development of AF. This study aims to investigate the clinical relationship between Neu5Ac and AF and left atrial enlargement.

**Methods:**

Forty-five patients with AF (AF group) and forty-five patients with non-AF (control group) matched for age, sex, and hospitalization date were recruited for our study. Plasma concentrations of Neu5Ac from peripheral venous blood were analyzed using enzyme-linked immunosorbent assay (ELISA). The baseline characteristics, plasma level of Neu5Ac, and echocardiographic characteristics were evaluated.

**Results:**

The plasma level of Neu5Ac was significantly higher in the AF group than in the control group (107.66 ± 47.50 vs 77.87 ± 39.09  ng/ml; *P* < 0.05); the left atrial diameters were positively correlated with the plasma Neu5Ac level (*R* = 0.255; *P* < 0.05). The plasma Neu5Ac level (*R* = 0.368; *P* < 0.05) and the left atrial diameters (*R* = 0.402; *P* < 0.05) were positively correlated with AF history times. Neu5Ac (odds ratio 1.018, 95% CI 1.003–1.032; *P* < 0.05) and the left atrial diameter (odds ratio 1.142, 95% CI 1.020–1.280; *P* < 0.05) were independent risk factors for AF in multivariate regression analysis.

**Conclusions:**

Serum Neu5Ac is associated with atrial fibrillation, and the mechanism may involve left atrial enlargement.

## 1. Introduction

Atrial fibrillation (AF) is the most common arrhythmia and the leading cause of cardiovascular morbidity and mortality in clinical practice worldwide. AF is associated with a five-fold increased risk of stroke and a three-fold increased incidence of congestive heart failure, as well as increased mortality. Hospitalization of patients with AF is also very common, accounting for approximately one-third of hospitalizations for cardiac rhythm disturbances [1–3]. Fundamental mechanisms governing the incidence and prevalence of AF, including molecular, electrophysiological, and structural changes, remain poorly understood [4]. Experimental and clinical data have indicated that the incidence and progression of AF are very complex pathophysiological processes involving a large number of significant players, including atrial enlargement, Ca^2+^ overload, inflammation, apoptosis, and fibrosis, autonomic nervous system changes [5–7].

The evidence from many of the studies has confirmed that sialic acids are involved in some pathological phenomena, such as inflammation, apoptosis, growth, and aging, and sialic acids are also involved in the binding and transport of Ca^2+^ [8]. N-Acetylneuraminic acid (Neu5Ac) is the most widespread form of sialic acid. Furthermore, recent studies have indicated that Neu5Ac plays a key role in severe coronary artery diseases, involving the activation of the RhoA signaling pathway, which is important for cardiac fibrosis [9–11]. Therefore, we speculated that Neu5Ac may be associated with AF and involved in the development of AF. We attempted to identify the association between them through this study.

## 2. Methods

### 2.1. Study Population

We recruited consecutive patients with AF (AF group) who were admitted to the Renmin Hospital of Wuhan University to schedule their first AF catheter ablation between March 2019 and July 2019, and consecutive patients with non-AF were selected to form a control group; these individuals were matched with the AF group for age, sex, and hospitalization date. After assessment of detailed medical history and a complete physical examination, some clinical examinations needed to be completed within 3 days after admission, such as biochemical testing, a 12-lead electrocardiogram (ECG), and echocardiograph. The history of AF was the period from the initial episode of AF to the time of admission.

The exclusion criteria were as follows: a history of prior AF catheter ablation, which might lead to atrial fibrosis and interfere with the results of study; atrial septal defect for occlusion or surgical treatment; severe coronary disease; heart valve disease; cardiac surgery, including heart transplantation and heart valve replacement; severe heart failure (might come with cardiac fibrosis which might interfere with the results of study); recent or active malignancy; severe renal failure (estimated glomerular filtration rate <30  ml/min); shock and death during hospitalization and unwillingness or inability to sign the informed consent form.

### 2.2. Biochemical Analysis

Venous blood samples were obtained from patients following overnight fasting (8 hours) after thirty minutes of rest in a sitting position before AF catheter ablation. Plasma concentrations of triglycerides, total cholesterol, low-density lipoprotein cholesterol (LDL-C), high-density lipoprotein cholesterol (HDL-C), creatinine, and uric acid were analyzed by an ADVIA 2400 Chemistry System (Siemens Healthcare Diagnostics, Munich, Germany).

### 2.3. Blood Sample Collection and Measurement of Neu5Ac

Venous blood samples were obtained from patients following overnight fasting (8 hours) after thirty minutes of rest in the sitting position before AF catheter ablation. A blood separator tube was used, and samples were allowed to clot for two hours at room temperature before centrifugation. Plasma samples were collected using a serum separator tube after centrifugation and then immediately frozen at −80°C until analysis. Later, the frozen serum samples were rapidly dissolved for analysis. Repeated freeze/thaw cycles were avoided. The plasma Neu5Ac level was evaluated with commercially available enzyme-linked immunosorbent assay (ELISA) kits according to the manufacturer's instructions (Human ELISA kit, Enzyme-linked Biotechnology, Shanghai, China).

### 2.4. Echocardiography

All patients underwent routine transthoracic echocardiography within 3 days after admission. Echocardiography was performed by an operator with over 5 years of experience using a GE E95 (GE Healthcare, Little Chalfont, United Kingdom). All measurements included at least 3 consecutive beats for patients. The left atrial diameter (LAD) was measured using a trailing edge-to-leading edge convention to measure the maximal distance between the posterior aortic root wall and the posterior left atrial wall at end ventricular systole. Left ventricular diameter and right ventricular diameter (normalized to body surface area) were measured at the end of ventricular diastole. The modified biplane Simpson rule was applied to calculate the left ventricular (LV) ejection fraction.

### 2.5. Statistical Analysis

All continuous variables are expressed as the mean ± SD for normally distributed data, and categorical variables are presented as counts (percentages). Independent-sample Student's *t*-test or chi-square test was used for comparisons of continuous and categorical variables, respectively. Correlation analysis was performed using Pearson's correlation analysis. Multivariate logistic regression analysis was used to identify risk factors. SPSS version 17.0 (SPSS, Chicago, Illinois) was used for data analysis. Differences were considered significant when a 2-sided *P* value was <0.05 for all the comparisons.

## 3. Results

### 3.1. Baseline Characteristics

Table 1 presents the baseline characteristics and the comorbidities of the study patients. Forty-five consecutive patients with AF were included in the AF group, and 45 patients with non-AF were matched with the AF group for age, sex, and hospitalization date as the control group. The left atrial diameters (LAD) were significantly larger in the AF group than in the control group (LAD 40.20 ± 5.92 vs 36.73 ± 5.20  mm; *P* < 0.05). There were no significant differences in the prevalence of smoking, hypertension, diabetes, right atrial diameters, left ventricular diameters, right ventricular diameters, or left ventricular ejection fraction between the AF group and the control group. There were no significant differences in the levels of plasma triglycerides, total cholesterol, LDL-C, HDL-C, creatinine, or uric acid between the two groups.

### 3.2. Analysis of Plasma Neu5Ac Level

**Figure 1:**
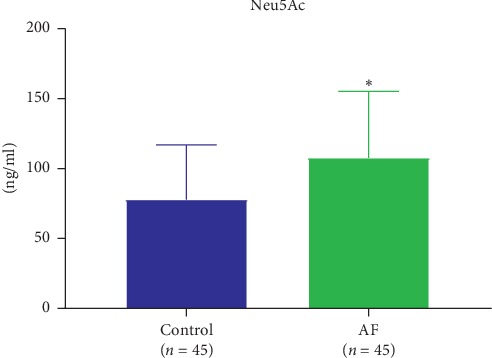
The plasma level of Neu5Ac was significantly increased in the AF group compared with the level in the control group.^*∗*^*p* < 0.05 for significant differences.

Figure 1 shows that the level of plasma Neu5Ac was significantly higher in the AF group than in the control group (107.66 ± 47.50 vs 77.87 ± 39.09  ng/mL; *P* < 0.05).

### 3.3. Multivariate Logistic Regression Analysis

The continuous variables were entered into the multivariate logistic regression analysis.  Table 2 shows that in the final multivariate logistic regression analysis model, plasma Neu5Ac (odds ratio 1.018, 95% CI 1.003–1.032; *P* < 0.05) and left atrial diameter (odds ratio 1.142, 95% CI 1.020–1.280; *P* < 0.05) were the independent risk factors for AF.

### 3.4. Correlation Analysis

**Figure 2:**
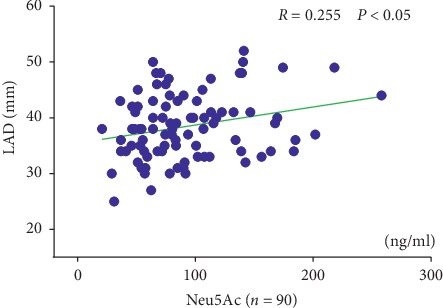
Correlation of plasma Neu5Ac levels with LAD. Neu5Ac: N-acetylneuraminic acid and LAD: left atrial diameter. *R* is Pearson's correlation coefficient.

Table 3 and Figure 2 show that the left atrial diameters were positively correlated with the plasma Neu5Ac level (*R* = 0.255; *P* < 0.05). There was no significant correlation between the plasma Neu5Ac level and the following factors: age, the level of plasma triglycerides, total cholesterol, LDL-C, HDL-C, creatinine, uric acid, and the right atrial, left ventricular, and right ventricular diameters.

**Figure 3:**
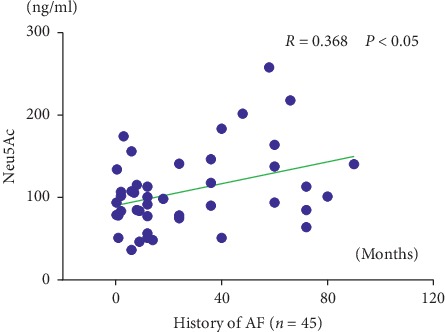
Correlation of the history of AF with Neu5Ac. Neu5Ac: N-acetylneuraminic acid. *R* is Pearson's correlation coefficient.

**Figure 4:**
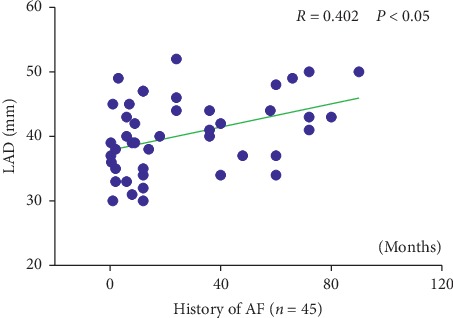
Correlation of the history of AF with LAD. LAD: left atrial diameter. *R* is Pearson's correlation coefficient.

Figures 3 and 4 show that the plasma Neu5Ac level (*R* = 0.368; *P* < 0.05) and the left atrial diameters (*R* = 0.402; *P* < 0.05) were positively correlated with the AF history times.

## 4. Discussion

Many studies have been performed in regard to the mechanism and therapeutic strategies of AF. Multiple factors, including advancing age, male sex, obesity, heart failure, obstructive sleep apnea, anxiety, systemic arterial hypertension, and biomarkers such as N-terminal pro-B-type natriuretic peptide (NT-proBNP), have been identified as factors correlated with AF development [12–15]. Our study showed that the level of plasma Neu5Ac was significantly higher in the AF group than in the control group, indicating that Neu5Ac was significantly correlated with AF.

All cells of every species in nature are covered by a dense and complex coating of glycans (oligosaccharides or polysaccharides). Some glycans are sialic acids. Neu5Ac is the most widespread form of sialic acid. Sialic acids are a family of neuraminic acid derivatives, sugars with a shared nine-carbon backbone that are typically found attached to the terminal positions of several amino groups of the cell surface, and secreted glycan molecules [16]. The amino groups with glycans are acetylated by the key catabolism enzyme sialidase, leading to Neu5Ac. Neu5Ac is found in the tissues of many mammals and is involved in a variety of pathophysiological functions [17].

Is Neu5Ac a risk factor or simply a marker or mediator molecule for AF? Our study indicated that LADs were positively correlated with the plasma Neu5Ac level. Previous studies have shown that LAD is associated with AF incidence and progression and is an important risk factor in the development of AF. LAD is also involved in the maintenance and recurrence of AF [18–20]. Therefore, we speculated that elevated plasma Neu5Ac levels may result in the enlargement of the left atrium, which facilitates the incidence and development of AF. Many factors are involved in left atrial enlargement, including AF itself. Left atrial enlargement can occur as a consequence of AF [21, 22]; therefore, the increase in the plasma Neu5Ac level may also be a consequence of AF and atrial cardiomyopathy.

The LAD sizes were also positively correlated with the AF history times in our study. Many factors are involved in left atrial enlargement, including AF itself. Left atrial enlargement can occur as a consequence of AF [21, 22]. Suzuki et al. found that LAD was associated with more AF days as well as with the progress and maintenance of AF [19]. Our study results also suggested that plasma Neu5Ac levels were positively correlated with AF history times. Therefore, we made a hypothesis that Neu5Ac may also be involved in the progression and maintenance of AF.

AF is a final common endpoint of atrial remodeling caused by a variety of cardiac diseases and conditions that contribute to the progression and maintenance of AF. Atrial remodeling includes structural remodeling and electrical remodeling. Structural remodeling is characterized by atrial enlargement and tissue fibrosis [23]. There is convincing evidence from many studies that myocardial fibrosis of the left atrium is involved in the pathophysiology of AF [24, 25]. Recent studies have indicated that serum Neu5Ac plays a key role in severe coronary artery diseases, involving RhoA signaling pathway activation through a molecular docking test [9]. It is well established that the RhoA signaling pathway is critically involved in cardiac fibrosis [11]. The Rho family of GTPases is thought to mediate the activation of the SRF-MRTF signaling pathway, which is directly related to cardiac fibrosis; furthermore, RhoA signaling is activated downstream of the TGF-*β* receptor [26, 27]. Studies have indicated an important role for TGF-*β* in both atrial fibrosis and atrial remodeling, involving the RhoA signaling pathway [28, 29]. Therefore, Neu5Ac may be involved in atrial fibrosis. Through the RhoA signaling pathway, elevated plasma Neu5Ac levels may result in fibrosis of the left atrium, which facilitates the progression and maintenance of AF.

## 5. Conclusions

Serum Neu5Ac is associated with atrial fibrillation. The elevated concentrations of plasma Neu5Ac may result in left atrial enlargement and fibrosis, which facilitate the development of AF.

## Figures and Tables

**Table 1:** Characteristics of the patients.

Characteristics	Control	AF	*p* value
Demographics			
No.	45	45	
Age (years)	61.02 ± 10.11	61.80 ± 11.68	0.74
Male, %	26 (57.8)	26 (57.8)	1.00
Smoking, %	7 (15.6)	8 (17.8)	0.78
Hypertension, %	25 (55.6)	19 (42.2)	0.21
Diabetes, %	6 (13.3)	8 (17.8)	0.63

Biochemical testing			
Triglycerides (mmol/L)	2.40 ± 1.56	2.04 ± 1.42	0.27
Total cholesterol (mmol/L)	4.43 ± 0.79	4.16 ± 0.88	0.14
LDL-C (mmol/L)	2.41 ± 0.62	2.23 ± 0.66	0.17
HDL-C (mmol/L)	1.15 ± 0.29	1.16 ± 0.32	0.85
Creatinine (*μ*mol/L)	73.32 ± 19.92	72.81 ± 17.03	0.90
Uric acid (*μ*mol/L)	397.00 ± 98.60	393.18 ± 102.72	0.86

Echocardiographic characteristics			
Left atrial diameter (mm)	36.73 ± 5.20	40.20 ± 5.92	<0.01^*∗*^
Right atrial diameter (mm)	35.18 ± 3.55	35.78 ± 4.44	0.48
Left ventricular diameter (mm)	44.07 ± 5.65	44.13 ± 3.98	0.95
Right ventricular diameter (mm)	20.87 ± 3.08	20.76 ± 2.42	0.85
LV ejection fraction (%)	57.82 ± 7.12	57.27 ± 4.88	0.68

LDL-C: low-density lipoprotein cholesterol; HDL-C: high-density lipoprotein cholesterol; LV: left ventricular. Values are mean ± SD or *n* (%). ^*∗*^*p* < 0.05 for significant differences.

**Table 2:** Multivariate logistic regression analysis of risk factors for atrial fibrillation.

	B	SE	Wald	*p*	OR	95% CI
Neu5Ac	0.017	0.007	5.896	0.015^*∗*^	1.018	1.003–1.032
Age	0.002	0.026	0.005	0.942	1.002	0.953–1.054
Triglycerides	−0.124	0.202	0.375	0.540	0.884	0.595–1.313
Total cholesterol	−0.535	0.356	2.263	0.133	0.586	0.292–1.176
LDL-C	−0.065	0.474	0.019	0.890	0.937	0.370–2.372
HDL-C	−0.122	1.005	0.015	0.903	0.885	0.123–6.349
Creatinine	−0.013	0.015	0.699	0.403	0.987	0.958–1.018
Uric acid	−0.002	0.003	0.467	0.494	0.998	0.992–1.004
LAD	0.133	0.058	5.303	0.021^*∗*^	1.142	1.020–1.280
RAD	0.039	0.065	0.354	0.552	1.039	0.915–1.180
LVD	−0.024	0.057	0.180	0.672	0.976	0.872–1.092
RVD	−0.073	0.130	0.316	0.574	0.930	0.721–1.199
LV ejection fraction	0.024	0.053	0.208	0.648	1.024	0.924–1.136

Neu5Ac: N-acetylneuraminic acid; LDL-C: low-density lipoprotein cholesterol; HDL-C: high-density lipoprotein cholesterol; LAD: left atrial diameter; RAD: right atrial diameter; LVD: left ventricular diameter; RVD: right ventricular diameter; LV: left ventricular. ^*∗*^*p* < 0.05 for significant differences.

**Table 3:** Correlation analysis with Neu5Ac.

	*R*	*p* value
History of AF	0.368	0.013^*∗*^
Age (years)	0.071	0.503
Triglycerides (mmol/L)	0.021	0.848
Total cholesterol (mmol/L)	0.054	0.619
LDL-cholesterol (mmol/L)	−0.055	0.608
HDL-cholesterol (mmol/L)	−0.060	0.588
Creatinine (*μ*mol/L)	0.098	0.364
Uric acid (*μ*mol/L)	0.084	0.435
Left atrial diameter (mm)	0.255	0.015^*∗*^
Right atrial diameter (mm)	−0.038	0.720
Left ventricular diameter (mm)	0.130	0.223
Right ventricular diameter (mm)	0.000	0.994

R is Pearson's correlation coefficient. Neu5Ac: N-acetylneuraminic acid; LDL-C: low-density lipoprotein cholesterol; HDL-C: high-density lipoprotein cholesterol; ^*∗*^*p* < 0.05 for significant differences.

## Data Availability

The data underlying the findings of the study are available on request to the corresponding author.

## Additional Points

There exist a few limitations in our study. First, the study group size was relatively small. Second, this study indicated that serum Neu5Ac was closely correlated with AF, but showed lack of powered cause-and-effect evidence. Third, LAD, LA area, and volume are all important parameters for the evaluation of left atrial enlargement. We only used LAD as a parameter of left atrial size rather than LA area and volume. Fourth, there was a lack of data on cardiac magnetic resonance or atrial voltage mapping with Carto or EP mapping systems to quantify atrial fibrosis.
